# Overcoming Clopidogrel Resistance in Carotid Artery Stenting: Experience with Ticagrelor and Ticlopidine

**DOI:** 10.3390/clinpract16020038

**Published:** 2026-02-10

**Authors:** Pavol Vigláš, Jan Raupach, Aleš Hejčl, David Černík, Pavla Bradáčová, Patrik Matras, Filip Cihlář

**Affiliations:** 1 Department of Radiology, J. E. Purkyně University—Faculty of Health Studies and Krajská zdravotní—Masaryk Hospital, Sociální péče 3316/12A, 401 13 Ústí nad Labem, Czech Republic; pavol.viglas@kzcr.eu (P.V.);; 2 Faculty of Medicine in Hradec Králové, Charles University, Sokolská 581, 500 05 Hradec Králové, Czech Republic; 3 Department of Radiology, University Hospital Hradec Kralove, Sokolská 581, 500 05 Hradec Králové, Czech Republic; 4 Department of Neurosurgery, J. E. Purkyně University—Faculty of Health Studies and Krajská zdravotní—Masaryk Hospital, Sociální péče 3316/12A, 401 13 Ústí nad Labem, Czech Republic; 5 Department of Neurology, Krajská zdravotní—Masaryk Hospital, Sociální péče 3316/12A, 401 13 Ústí nad Labem, Czech Republic; 6 Department of Neurology, Palacký University Medical School and University Hospital, Zdravotníků 248/7, 779 00 Olomouc, Czech Republic; 7 Department of Clinical Heamatology, Krajská zdravotní—Masaryk Hospital, Sociální péče 3316/12A, 401 13 Ústí nad Labem, Czech Republic; 8 First Faculty of Medicine, Charles University, Kateřinská 1660/32, 121 08 Praha, Czech Republic

**Keywords:** antiplatelet therapy, clopidogrel, ticagrelor, carotid stenting

## Abstract

**Objectives:** The goal of this study is to establish the incidence of high on-treatment platelet reactivity (HTPR) to aspirin and clopidogrel in patients undergoing carotid stenting and to evaluate the efficacy of ticagrelor and ticlopidine in patients with HTPR to clopidogrel. **Methods:** In a single institutional setting spanning eight years, every consecutive patient who underwent carotid artery stenting was incorporated into a study. Subsequently, a retrospective analysis of their platelet function was executed. Prevalence of high on-treatment reactivity to aspirin, clopidogrel, ticlopidine and ticagrelor was assessed. Platelet function testing was conducted by light transmission aggregometry and Multiplate^®^. **Results:** A total of 216 patients were tested for antiplatelet therapy efficacy. The high on-treatment reactivity to clopidogrel was observed in 68 patients (31.4%). No patients with high on-treatment reactivity to ticagrelor or ticlopidine were observed. There was a significant reduction in platelet reactivity with ticagrelor (*p* < 0.000) and ticlopidine (*p* < 0.000) in patients with HTPR to clopidogrel. **Conclusions:** High on-treatment platelet reactivity to clopidogrel is common in patients undergoing carotid artery stenting. Ticagrelor is a viable alternative to overcome HTPR to clopidogrel. These findings suggest that platelet function testing can identify patients who may benefit from tailored antiplatelet therapy in reducing thromboembolic complications after carotid stenting.

## 1. Introduction

Carotid artery stenting (CAS) has become an established and widely accepted treatment option for carotid artery stenosis, representing an important alternative to surgical revascularization. The importance of CAS lies in secondary stroke prevention in symptomatic patients along with best medical treatment (BMT). BMT consists of antiplatelet agents, statins, blood pressure control, smoking cessation, and a healthy lifestyle [1]. Large, randomized control trials proved non-inferiority to carotid endarterectomy (CEA) with comparable periprocedural outcomes, long-term stroke prevention, and durability [2,3,4,5,6,7]. The spectrum of periprocedural complications distinguishes carotid endarterectomy (CEA) from carotid artery stenting (CAS). Specifically, myocardial infarction and cranial nerve paresis are more frequently associated with surgical intervention, whereas thromboembolic events are observed more often following endovascular treatment [8,9,10,11].

Dual antiplatelet therapy (DAPT) therefore plays a crucial role in preventing thromboembolic complications. Dual antiplatelet therapy (DAPT) is formulated with acetyl-salicylic acid and an additional pharmaceutical agent classified as a P2Y12 receptor inhibitor. The most frequently prescribed combination involves acetyl-salicylic acid (ASA) and clopidogrel [1,2]. Despite this therapy, the risk of periprocedural thromboembolic complications in some patients persist. The increased risk of thromboembolic complications is associated not only with differences in the characteristics of the instrumentation used, but also in interindividual variability in response to antiplatelet therapy, particularly in the case of clopidogrel. The variability in clopidogrel effectiveness mainly arises from genetic polymorphism and potential interactions with other medications. The presence of comorbidities further diminishes the treatment’s efficacy [12,13]. This leads to a phenomenon known as high on-treatment platelet reactivity (HTPR) [12,13,14,15]. Patients with HTPR can also be referred to as non-responders, low-responders or resistant. Clinically, HTPR can be defined as a failure to prevent thromboembolic events, which can also be referred to as treatment failure [13,16]. Antiplatelet drug HTPR is well-documented and has been associated with thromboembolic complications in patients after coronary stent placement [17,18]. Multiple studies in recent years have reported the incidence of clopidogrel HTPR in cohorts of patients undergoing neuro-endovascular procedures, as well as a higher incidence of thromboembolic complications in these patients [19,20,21,22,23,24]. The incidence of clopidogrel HTPR in the literature varies from 21% to 53% in patients undergoing neuro-endovascular procedures, but evidence of incidence of HTPR in patients undergoing CAS is limited [12,23,25]. The current literature suggests a potential link between clopidogrel HTPR and thromboembolic complications in patients who underwent CAS [23,24]. The routine use of platelet function testing before neurovascular procedures with stent implantation is currently a topic of significant debate within the neurointerventional community, and it is not considered to be part of the standard of care in most centers [24]. Recent studies have described the utility of platelet function testing within endovascular patients undergoing intracranial procedures, but very few studies have focused exclusively on patients undergoing extracranial carotid artery stenting procedures [24,26,27,28]. Preprocedural testing for clopidogrel HTPR in CAS could be beneficial in terms of reducing thromboembolic complications [23,24,29].

## 2. Materials and Methods

In this retrospective, single-center observational study, we investigated platelet reactivity in all consecutive patients undergoing carotid stenting at Masaryk Hospital, Czech Republic (MNUL) from 2015 to 2024. The goal of this study is to determine the incidence of high on-treatment platelet reactivity to standard dual antiplatelet therapy with aspirin and clopidogrel, and to evaluate the effectiveness of tailored antiplatelet therapy (ticagrelor, ticlopidine) in patients with high on-treatment platelet reactivity to clopidogrel. Clinical outcomes of tailored antiplatelet therapy in carotid stenting were published elsewhere (https://cvirendovasc.springeropen.com/articles/10.1186/s42155-024-00482-2) (accessed on 10 November 2025).

### 2.1. Study Criteria

Patients diagnosed with carotid stenosis who underwent carotid stenting were included in this study. Stenosis severity was assessed utilizing the criteria established by the North American Symptomatic Carotid Endarterectomy Trial (NASCET). Carotid artery stenting procedures were conducted when symptomatic individuals presented with stenosis ranging from 50% to 99%, and for asymptomatic patients exhibiting stenosis, levels were between 70% and 99%. The decision to perform carotid artery stenting (CAS) was made subsequent to a multidisciplinary consensus and a comprehensive evaluation of high-risk anatomical and procedural characteristics, specifically encompassing arterial problems, tortuosity, challenges in achieving secure vascular access, and the presence of a circumferentially calcified lesion. All included patients were considered high-risk for surgery. Both symptomatic and asymptomatic carotid stenosis cases were included. The evaluation of clinical manifestations was conducted by the referring neurologist or neurosurgeon. Individuals demonstrating evidence of cerebral ischemia or amaurosis fugax within the preceding six months were designated as symptomatic patients. Primary stenosis and restenosis after carotid endarterectomy were included, while other indications for carotid stenting, such as carotid dissection, were excluded. Patients with unavailable results of platelet function tests for even one drug of DAPT were excluded. In these cases, the test was performed outside the hospital, and effective antiplatelet therapy was established in an outpatient clinic. Patients with thrombocytopenia (platelet count < 100 × 10^3^/μL) within 30 days before testing were also excluded. Each patient was tested using either light transmission aggregometry or the Multiplate^®^ method, but never both. Patients who were initially tested with one method and then adjusted their therapy and tested with the other method were excluded.

### 2.2. Antiplatelet Therapy Regime

The standard DAPT regimen with aspirin and clopidogrel had to be initiated at least five days before platelet function testing, using doses of 100 mg of aspirin and 75 mg of clopidogrel. If clopidogrel HTPR was found, treatment was changed to ticlopidine, which was taken for a minimum of five days before the repeated antiplatelet therapy test at a dose of 2 × 250 mg daily. Subsequent to the market discontinuation of ticlopidine in 2020, ticagrelor became its successor. The protocol for ticagrelor involved the administration of a 2 × 90 mg loading dose, followed by a repeat platelet function assessment on the subsequent day. The administration of DAPT was advised for a duration of no less than one month following the procedure, incorporating daily maintenance doses of 100 mg ASA in conjunction with 75 mg clopidogrel, or 90 mg ticagrelor twice daily. ASA monotherapy was continued after the completion of DAPT. In patients with atrial fibrillation receiving chronic oral anticoagulation, the oral anticoagulation was temporarily withheld peri-procedurally and reinitiated after carotid artery stenting. Long-term triple antithrombotic therapy was avoided.

### 2.3. Platelet Function Tests Details

Light transmission aggregometry (LTA), a long-standing and well-validated technique for evaluating platelet functionality, was conducted using a turbidimetric approach on an APACT 4004 aggregometer (LAbor BioMedical Technologies, Ahrensburg, Germany) [19,20,22]. Blood samples, which were prevented from clotting by collection into 0.109 M sodium citrate at a 1:9 ratio, underwent initial centrifugation at 150× *g* for 10 min to yield platelet-rich plasma (PRP). Platelet-poor plasma (PPP) for blank measurements was prepared by centrifuging a portion of the sample at 2500× *g* for 10 min. The optimal platelet concentration in PRP was established between 150 and 600 × 10^9^/L; counts exceeding 600 × 10^9^/L were adjusted to 350 × 10^9^/L with saline, while counts below 150 × 10^9^/L necessitated a cautionary note regarding potential artifacts in aggregation results. Aggregation was induced by adding either 4 µmol/L adenosine diphosphate (ADP) or 1 mmol/L arachidonic acid (ACA) to 140 µL of citrated PRP, maintained at 37 °C with continuous stirring at 250× *g*. The formation of platelet aggregates resulted in increased light transmission through the PRP as it clarified, with these changes continuously monitored over a 10 min period and recorded as an aggregation curve. Results were expressed as the maximum amplitude percentage or maximum of aggregation (MoA), with antiplatelet therapy efficacy, specifically for acetyl-salicylic acid using ACA as an inducer, defined as an MoA ranging from 0 to 20; P2Y12 inhibitors were in vitro defined as MoA ≥ 60% with 4 umol of ADP agonist used [20]. There was a gray zone of MoA = 60% ± 5% where the clinical efficacy of antiplatelet therapy was unclear and a decision to adjust therapy was made after the complex evaluation of platelet function including aspirin efficacy.

The Multiplate^®^ analyzer (Roche Diagnostics, Mannheim, Germany) determines platelet aggregation through impedance alteration measurements. The analytical process involved transferring 1.6 mL of blood into a hirudin-coated tube (Monovette-S, Sarstedt, Nümbrecht, Germany), which was then allowed to equilibrate at ambient temperature. Platelet inhibition was assessed using the ASPI and ADP assays, conducted with the Multiplate Analyzer, in accordance with the manufacturer’s specifications. Initially, 300 µL of hirudin-anticoagulated blood was diluted with prewarmed (37 °C) isotonic sodium chloride. After a 3 min incubation period at 37 °C, 20 µL of the appropriate agonist, either arachidonic acid or adenosine diphosphate (ADP), was introduced into each sample. Exclusively commercially available standard reagents from Roche Diagnostics (Mannheim, Germany) were employed. The active measurement phase for each assay spanned 6 min. The observed aggregation was quantified by aggregation units [AUs], aggregation velocity [AU/min], and the area under the curve (AUC, AU ∗ min[U]). For subsequent data interpretation, the AUC was chosen as the primary output metric. The use of a hirudin-coated tube established specific thresholds of < 45 U for ADP and < 30 U for the ASPI test. The gray zone of 45 ± 5 U was defined, where the complex evaluation of platelet function including aspirin efficacy had to be made before adjusting the therapy. In some cases, a past platelet function test result was beneficial. The testing location was standardized.

### 2.4. Statistical Analysis

The demographic variables were summarized utilizing descriptive statistics, with presentations as either mean standard deviation or counts (percentages). For categorical variables, counts and percentages were used for summarization, and differences were evaluated via the χ^2^ test. The Kolmogorov–Smirnov test confirmed the normality of the data. Further statistical analysis incorporated the Fisher exact test and the test for the difference in means.

*p* values < 0.05 were considered statistically significant. STATISTICA 13.3 software was used for statistical analysis.

## 3. Results

During the designated period, the carotid artery stenting was performed in 256 patients. Of the 256 patients who underwent carotid artery stenting during the study period, 36 were excluded due to incomplete platelet function testing or protocol deviations, resulting in a final study cohort of 216 patients.

In the study population, primary atherosclerotic stenosis was identified in 184 carotid arteries (83.6%), with carotid restenosis after prior carotid endarterectomy (CEA) observed in 36 cases (16.4%). Clinical intervention addressed symptomatic stenosis in 120 carotid arteries (54.5%) and asymptomatic stenosis in 100 carotid arteries (45.5%). The mean age of the participants was 71.2 years, with ages ranging from 45 to 93 years. The most prevalent comorbidity throughout the cohort was arterial hypertension, affecting 73.4% of patients, followed by dyslipidemia in 63.4%. Additionally, 36.4% of patients were undergoing treatment for diabetes mellitus, and half of the patients reported current or past active smoking. Detailed demographic information and comorbidity profiles are presented in Table 1.

There was a significant difference in the incidence of hypertension, diabetes mellitus and atrial fibrillation between patients with HTPR to clopidogrel and patients with appropriately lower platelets reactivity.

A platelet function test was performed using light transmission aggregometry in 62.7% (*n* = 138) and by Multiplate^®^ in 37.3% (*n* = 82). All patients (*n* = 216) had DAPT with clopidogrel prior platelet function testing.

### 3.1. HTPR to Acetyl-Salicylic Acid

Overall, HTPR to ASA was found in 56 patients (25.4%). Using the LTA method, the MoA was 22 ± 22.9% in patients with ASA therapy (*n* = 141). In a subgroup of non-responders to ASA (*n* = 32), mean MoA was 55.1 ± 27.1% and in a subgroup of responders (*n* = 109) mean MoA was 12.3 ± 7.0%.

Patients tested with the Multiplate method had mean 25.4 ± 26.3 U in the ASPI test (*n* = 79). The subgroup of non-responders to ASA had mean 53.9 ± 36.2 U (*n* = 22). The subgroup of responders had mean 14.6 ± 6.6 U (*n* = 57).

### 3.2. HTPR to Clopidogrel

Incidence of HTPR to clopidogrel was 31.4% (*n* = 68/216).

In all patients with clopidogrel therapy the mean MoA was 61.0 ± 21.1% (*n* = 136), in a subgroup of responders the mean MoA was 51.8 ± 17.4% (*n* = 89) using the LTA method. In the subgroup of patients resistant to clopidogrel (*n* = 47) the mean MoA was 78.9 ± 15.4%. Thirty-four clopidogrel-resistant patients identified by LTA (the mean MoA 78.5 ± 16.6%, min. MoA 60%, max MoA 121%) were converted to ticagrelor. Thirteen patients resistant to clopidogrel with the mean MoA 84 ± 8.3% (minimum MoA was 70%, maximum 98%) were converted to ticlopidine therapy.

The mean results of the Multiplate method in patients with clopidogrel was 31.9 ± 26.0 U (*n* = 80), and the responders to clopidogrel (*n* = 60) had a mean 20.7 ± 8.1 U. The 11 patients resistant to clopidogrel, who were tested by Multiplate, and were later converted to ticagrelor, had a mean 92.7 ± 33.8 U (min. 64 U, max. 121 U). Ten patients resistant to clopidogrel with a mean MoA 48.2 ± 8.6 U, min. 41 U, max. 67 U were converted to ticlopidine. A total of 29 patients (13.1%) were resistant to both aspirin and clopidogrel.

### 3.3. HTPR to Ticagrelor

Out of 68 patients with HTPR to clopidogrel, P2Y12 inhibitor in DAPT was changed to ticagrelor 45 times (66.2%).

**Figure 1:**
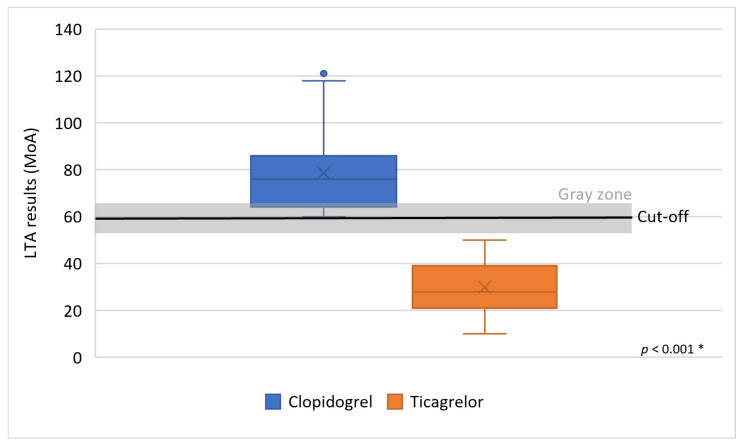
Effect of ticagrelor in patients resistant to clopidogrel using light transmission aggregometry. Legend: LTA—light transmission aggregometry, MoA—maximum of aggregation, *—groups were compared using the Mann–Whitney U test.

Thirty-four clopidogrel-resistant patients identified by LTA were converted to ticagrelor with a significant reduction in platelet reactivity to the mean MoA 29.7% ± 10.5%; the maximum MoA was 50%, and the minimum MoA 10% (*p* < 0.001) (Figure 1). No patients with HTPR to ticagrelor were observed.

**Figure 2:**
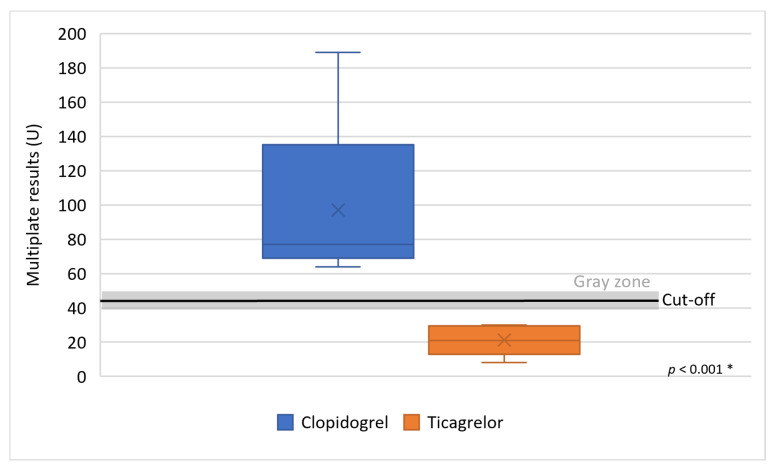
Effect of ticagrelor in patients resistant to clopidogrel using Multiplate method. Legend: U—area under the curve, *—groups were compared using the Mann–Whitney U test.

Eleven clopidogrel-resistant patients by Multiplate showed, after conversion to ticagrelor, a mean 21.2 ± 8.9 U (min. 8 U, max. 30 U), which was statistically significant, *p* < 0.001 (Figure 2).

### 3.4. HTPR to Ticlopidine

Out of 68 patients with HTPR to clopidogrel, P2Y12 inhibitor in DAPT was changed to ticlopidine 23 times (33.8%).

**Figure 3:**
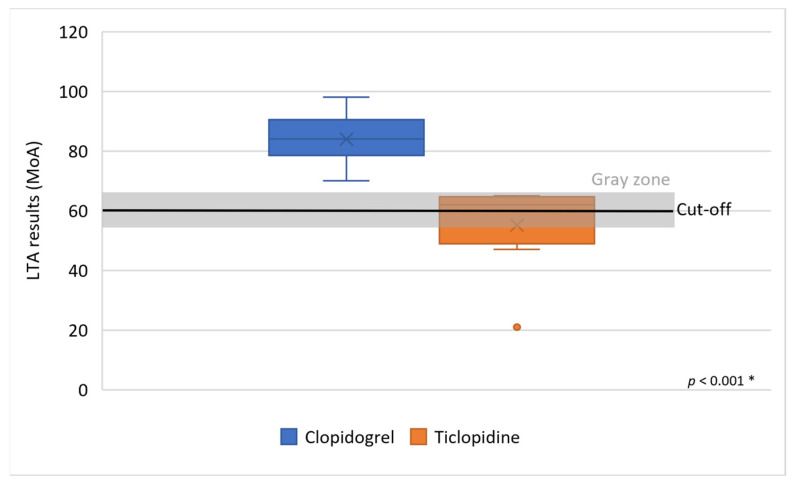
Effect of ticlopidine in patients resistant to clopidogrel using light transmission aggregometry. Legend: MoA—maximum of aggregation, *—groups were compared using the Mann–Whitney U test.

Thirteen patients resistant to clopidogrel identified by LTA were converted to ticlopidine therapy, which significantly reduced platelet reactivity to the mean MoA 55.1 ± 15.1% (maximum MoA was 65%, minimum MoA was 21%) (*p* < 0.001) (Figure 3). No patient with HTPR to ticlopidine (mean MoA was 58.6 ± 17.1%) was observed.

**Figure 4:**
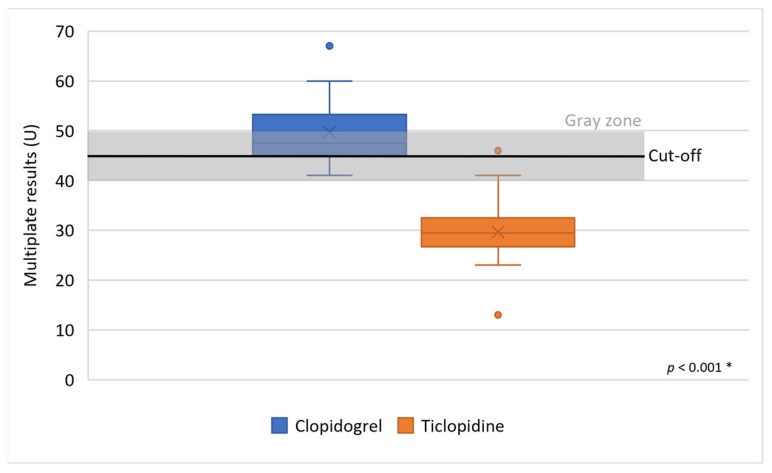
Effect of ticlopidine in patients resistant to clopidogrel using Multiplate method. Legend: U—area under the curve, *—groups were compared using the Mann–Whitney U test.

Ten patients resistant to clopidogrel identified by the Multiplate method were converted to ticlopidine (mean MoA 29.7 ± 7.7U, min. 13 U, max. 46 U). The difference was also significant, *p* < 0.001 (Figure 4).

## 4. Discussion

This was a retrospective study in which platelet function testing was performed before the carotid stenting. Our results are comparable to the incidence of HTPR to clopidogrel published in studies with patients undergoing intracranial neuro-endovascular procedures. HTPR to clopidogrel ranging from 21% to 53% has been described in the literature [12,15,19]. In our study, the incidence of HTPR in patients undergoing carotid stenting was found in 31.4% of patients. All 68 patients with HTPR to clopidogrel were responders to the antiplatelet therapy change. We have recorded no patients with HTPR to ticagrelor or ticlopidine. Since ticlopidine is no longer in clinical use, ticagrelor still showed itself to be a viable alternative to overcome HTPR to clopidogrel and eventually reduce thromboembolic complications during carotid stenting.

Briefly, in our previously published study, tailored antiplatelet therapy guided by platelet function testing was associated with a significantly lower rate of periprocedural stroke or death compared with outcomes reported in large randomized carotid stenting trials CREST (Stenting versus Endarterectomy for Treatment of Carotid Artery Stenosis) and ICSS (The International Carotid Stenting Study), without an increase in major bleeding complications. In total, only five thromboembolic complications (2.01%) occurred and four of them were procedure-related. Two patients died because of procedure-related stroke [29].

Ticlopidine was included in this study only during the early phase of the study period, before its market discontinuation and before ticagrelor became routinely available. Although ticlopidine demonstrated adequate platelet inhibition in our cohort, its clinical use has largely been abandoned due to well-documented safety concerns, including neutropenia, thrombotic thrombocytopenic purpura, and hepatic toxicity. For these reasons, ticlopidine should not be considered a preferred alternative in contemporary practice. In contrast, ticagrelor offers rapid, potent, and predictable platelet inhibition with a more favorable safety profile, and our findings support its use as the primary alternative agent for overcoming clopidogrel resistance in patients undergoing carotid artery stenting.

Although laboratory-defined high on-treatment platelet reactivity to aspirin was observed in a subset of patients (25.4%), aspirin platelet function testing is not routinely recommended to guide therapy in contemporary vascular practice. In the present study, aspirin testing was performed for comprehensive platelet function assessment and contextual interpretation of P2Y12 inhibition rather than to identify clinically actionable aspirin resistance. Consequently, aspirin HTPR findings should be interpreted cautiously and without direct clinical implication.

Treating carotid artery stenosis mandates a comprehensive approach involving optimal pharmacotherapy, such as antiplatelet agents, statins, and antihypertensives, complemented by a healthy lifestyle and the cessation of smoking [23,24,29]. From our perspective, optimal antiplatelet management prior to an intervention is considered as crucial as accurate patient stratification and the secure execution of the procedure. American Heart Association (AHA) guidelines now clearly recommend a preference for newer, more potent antiplatelet agents such as ticagrelor and prasugrel in the treatment of acute coronary syndrome. The use of clopidogrel should then be limited to situations where other treatments are contraindicated or unavailable [30]. A recent meta-analysis of patients undergoing coronary intervention demonstrated better outcomes in patients with DAPT tailored by platelet function testing or genetic testing. At the same time, there was no increased rate of bleeding compared with patients with standard DAPT [31]. Multiple studies have shown that tailored antiplatelet therapy reduces the risk of thromboembolic complications, especially during intracranial aneurysm treatment with stenting and carotid stenting [21,32]. Similar clinical outcome was found in our study publisher earlier [29]. Patient adherence and compliance with antiplatelet therapy represent important factors that may influence platelet reactivity and clinical outcomes. All patients received standardized instructions regarding antiplatelet therapy initiation, and medication use was verified at hospital admission. In addition, platelet function testing itself provided an indirect assessment of effective drug intake. In one non-compliant patient, an acute stent thrombosis followed with major stroke was observed in previously published data [29].

Our study has several limitations, particularly the retrospective design of the study. This study is descriptive in nature, and no multivariable regression analysis was performed to identify independent predictors of HTPR. Therefore, associations between clinical characteristics and platelet reactivity should not be interpreted as causal. Another limitation is the methodological setting of cut-off values, as there is a lack of standardization between laboratories and the use of antiplatelet therapy in interventional neuroradiology varies considerably between centers. Another limitation is that, in the case of clopidogrel, a single test is very likely not sufficient to exclude the effect of intraindividual variation in efficacy levels. The correlation between LTA results and the newer Multiplate^®^ and VerifyNow^®^ methods is also problematic at present, complicating efforts to detect HTPR to antiplatelet therapy in a more accessible, standardized and rapid manner. Many authors refer to LTA as the gold standard in assessing the efficacy of antiplatelet therapy [12,33,34,35,36]. This is because it provides a comprehensive assessment of platelet function in the diagnosis of multiple platelet-related or bleeding disorders [12]. At the same time, the authors acknowledge methodological differences that may lead to different prevalence rates of “resistant” patients. The main differences include the dose of the agonist used, which is usually from 5 to 20 µmol/L for ADP and 1 mmol/L for ACA, the type of anticoagulant [citrate or hirudin], and the cut-off value of the LTA result in % determining the HTPR threshold. Standard values have not yet been established [37]. Different studies [when using an ADP agonist] use a platelet inhibition HTPR threshold from ≥ 46 to ≥ 80% [33,38]. In our laboratory, the cut-off for HTPR was set at ≥ 60%, which was also used by other studies [38]. Raising the threshold for efficacy to 70% or 80% would increase the number of patients with effective treatment. The LTA method is also used as a reference to newer methods such as Multiplate [Roche Diagnostics], Platelet Works [Helena Laboratories] or IMPACT-R [Daned SA] [12,21,34]. Other authors do not recommend the use of LTA precisely because of the lack of standardization between laboratories and the time-consuming preparation process [12]. Ideally, testing should be rapid, requiring a small blood sample volume, with simple handling, and correlating well with LTA. This can be fulfilled by the semiautomated Multiplate test, which is more reproducible. Another alternative may be the automated VerifyNow method [12,34]. However, it should be emphasized that, in the work of Flechtenmacher comparing all three methods, their agreement was only partial. In that study, the clopidogrel HTPR was around 50% and LTA results correlated with thromboembolic complications more than VerifyNow and Multiplate [37]. Several studies have shown that the individual response to clopidogrel is not uniform in all patients and is subject to inter- and intraindividual variability [36,39,40,41]. It is because of intraindividual variability that assessment of clopidogrel efficacy based on a single test may be unreliable [39,40]. Moreover, several studies have shown that the efficacy rate of its treatment decreases over time [39,40]. Like most studies, we evaluated the efficacy of antiplatelet therapy based on a single test. Especially at borderline efficacy values, a decision to adjust therapy was made after the complex evaluation of platelet function, including aspirin efficacy. Our results confirmed the interindividual variability of response to clopidogrel. In our study, HTPR to aspirin was found in 24.5% patients undergoing carotid stenting compared with those who were not. Other authors, based on various laboratory definitions for HTPR, report incidence HTPR to aspirin from 2.1% to 13.5% [12,33,40]. Our research confirmed that ticagrelor has a significantly reduced incidence of HTPR, indicating that it serves as a suitable alternative to clopidogrel. Using both LTA and the Multiplate method, we confirmed a high efficacy (100%) of ticagrelor treatment. Routine assessments encompassed the testing of ASA’s capacity to provide a complete understanding of platelet functionality. ASA is presently incorporated as a constant element within dual antiplatelet therapy. Empirical evidence thus far has not indicated that increased ASA concentrations diminish the incidence of thromboembolic events. Additionally, no substitute pharmaceutical exists to replicate the therapeutic effects of ASA [42]. Consequently, our attention was exclusively directed towards substitute medications for clopidogrel.

## 5. Conclusions

Resistance or high on-treatment platelet reactivity (HTPR) to clopidogrel is common among patients undergoing carotid artery stenting. In total, 31.4% of patients were resistant to clopidogrel. Switching to ticagrelor or ticlopidine therapy resulted in a complete normalization of platelet reactivity, with no cases of HTPR to these alternative agents. These findings support the use of platelet function testing to guide antiplatelet therapy in carotid interventions. Tailored treatment with ticagrelor appears to be an effective strategy to overcome clopidogrel HTPR and may help reduce thromboembolic complications. Prospective, multicenter studies are warranted to confirm these results and establish standardized platelet function testing protocols.

## Figures and Tables

**Table 1:** Demographic characteristics of the patient population.

Characteristic	HTPR to Clopidogrel	Clopidogrel Effective	*p* Value
No. of patients	68	148	
Mean age ± SD, years	71.9 ± 8.0	71.0 ± 9.1	0.497
BMI	29.8 ± 7.5	27.2 ± 7.4	0.151
Sex			0.545
Male	50 (73.5)	115 (77.7)	
Female	19 (27.9)	33 (22.3)	
Coronary artery disease	22 (32.3)	38 (25.6)	0.303
Hypertension	58 (85.2)	100 (67.5)	0.008
Diabetes mellitus	37 (54.4)	43 (29.0)	<0.001
Chronic kidney disease	6 (8.82)	7 (4.7)	0.270
Smoking	36 (52.9)	73 (49.3)	0.624
Dyslipidemia	49 (72.0)	90 (60.8)	0.112
Atrial fibrillation	14 (20.5)	12 (8.1)	0.014
History of ischemic stroke	39 (57.3)	82 (55.4)	0.078

Legend: (*n* (%)), SD—standard deviation.

## Data Availability

The raw data supporting the conclusions of this article will be made available by the authors on request.

## Abbreviations

The following abbreviations are used in this manuscript:

CAS	Carotid artery stenting
DAPT	Dual antiplatelet therapy
ASA	Acetyl-salicylic acid
HTPR	High on-treatment platelet reactivity
LTA	Light transmission aggregometry
